# Genomic Profiling Identifies Novel Mutations and SNPs in *ABCD1* Gene: A Molecular, Biochemical and Clinical Analysis of X-ALD Cases in India

**DOI:** 10.1371/journal.pone.0025094

**Published:** 2011-09-22

**Authors:** Neeraj Kumar, Krishna Kant Taneja, Veena Kalra, Madhuri Behari, Satinder Aneja, Surendra Kumar Bansal

**Affiliations:** 1 Department of Biochemistry, Vallabhbhai Patel Chest Institute, University of Delhi, Delhi, India; 2 Functional Genomics Unit, Institute of Genomics and Integrative Biology, Delhi, India; 3 Department of Pediatrics, All India Institute of Medical Sciences, New Delhi, India; 4 Department of Neurology, All India Institute of Medical Sciences, New Delhi, India; 5 Department of Pediatrics, Lady Hardinge Medical College and associated Kalawati Balsaran Children Hospital, University of Delhi, Delhi, India; National Institutes of Health, United States of America

## Abstract

X-linked adrenoleukodystrophy (X-ALD) affects the nervous system white matter and adrenal cortex secondary to mutations in the *ABCD1* gene that encode the peroxisomal membrane protein. We conducted a genomic and protein expression study of susceptibility gene with its clinical and biochemical analysis. To the best of our knowledge this is the first preliminary comprehensive study in Indian population that identified novel mutations and SNPs in a relatively large group. We screened 17 Indian indigenous X-linked adrenoleukodystrophy cases and 70 controls for mutations and SNPs in the exonic regions (including flanking regions) of *ABCD1* gene by direct sequencing with ABI automated sequencer along with Western blot analysis of its endogenous protein, ALDP, levels in peripheral blood mononuclear cells. Single germ line mutation was identified in each index case in *ABCD1* gene. We detected 4 novel mutations (2 missense and 2 deletion/insertion) and 3 novel single nucleotide polymorphisms. We observed a variable protein expression in different patients. These findings were further extended to biochemical and clinical observations as it occurs with great clinical expression variability. This is the first major study in this population that presents a different molecular genetic spectrum as compared to Caucasian population due to geographical distributions of ethnicity of patients. It enhances our knowledge of the causative mutations of X-ALD that grants holistic base to develop effective medicine against X-ALD.

## Introduction

X-linked adrenoleukodystrophy (X-ALD; OMIM # 300100) is one of the most frequent monogenic inherited peroxisomal neurodegenerative disorders. It affects the cerebral white matter, peripheral nerves, adrenal cortex and testis [1]. It is a recessive, usually male lethal, serious and progressive genetic disorder characterized by abnormal accumulation of saturated very long chain fatty acids (VLCFA) in body fluids and affected tissues, most notably in the brain and adrenal cortex due to an impaired β-oxidation in peroxisomes [1]–[4]. The frequency of X-ALD in USA is 1∶21,000 in males [5]. Seven different phenotypes have been described for male and five for female patients [6]. The more frequent male clinical phenotypes are cerebral form and adrenomyeloneuropathy (AMN). The cerebral forms viz. childhood, adolescent, and adult are characterized by intellectual, behavioral, cognitive, visual, gait disturbances associated with a rapid neurological progression, inflammatory reaction in the cerebral white matter and increased expression of cytokines which may involve autoimmune mechanisms [7], [8]. AMN, present only in adults, is characterized by gait, sensory, autonomic disturbances with slower progression, inflammatory response absent or mild and may affect the spinal cord and peripheral nerves. About 35% of AMN patients develop cerebral involvement at a later stage [6], [7]. Other less frequent phenotypes include asymptomatic, olivo-ponto-cerebellar (OPC) and Addison-only disorders (6). These different phenotypes might appear within the same family due to an identical *ABCD1* gene mutation [9]–[12], [24]. Additional genetic or environmental/epigenetic/stochastic factors have been suggested as the modulator of clinical phenotypes due to lack of any established genotype-phenotype correlation [13]–[15]. Moreover, the accurate molecular events from metabolic phase, characterized by cytoplasmic accumulation of VLCFA, to neuroinflammation and demyelination or axonal degeneration are still obscure. However, there are a few therapeutic approaches are under evaluation to control the devastating course of this disease. For many years dietary treatment based on a mixture of glyceroyl trioleate and glyceroyl trierucate (Lorenzo's oil) has been practiced as a preventive therapy [16]. Hydrocortisone replacement threapy was found to be useful in adrenal insufficiency X-ALD patients [17]. The progression of this disease was successfully halted by allogeneic hematopoietic cell transplantation (HCT) [18]. Recently, lentiviral-mediated gene therapy of hematopoietic stem cells was reported to provide clinical benefits in X-ALD patients [19].

The *ABCD1* (ATP-binding cassette subfamily D, member 1) gene defective in X-ALD was mapped to Xq28 [20]. It is 21 kb long gene, composed of 10 exons and codes for mRNA of 4.3 kb that is finally translated into 745 amino acid long peroxisomal ABC transporter adrenoleukodystrophy protein (ALDP), [21]. The imperfect ALDP leads to accumulation of saturated very long chain fatty acids (VLCFAs) in cytoplasm, which have to be exclusively catabolized by peroxisomal β-oxidation [22]. The biochemical diagnosis of X-ALD patients and carriers is based on the elevated level of VLCFA in plasma [23]. However, in 0.1% of affected males, the plasma C26:0 level is at borderline of the healthy subjects and 15% female heterozygotes have normal levels of VLCFA [24]. Molecular analysis with mutation detection is the only effective and reliable method to unambiguous determination of the genetic status of each individual at risk and to accurately rule out carrier status in females [25]. In the present study, we examined the mutations and SNPs in *ABCD1* gene in 17 patients with adrenoleukodystrophy, including 2 carrier females and 70 controls and report here the full spectrum of molecular defects of these patients describing the clinical features related to *ABCD1* gene mutations.

## Materials and Methods

### Patient Selection

The study included 17 patients of Indian origin (northern, southern and north-west region) attending the Department of Pediatrics and Department of Neurology, AIIMS, New Delhi or Department of Pediatrics, Lady Hardinge Medical College, University of Delhi, Delhi. The study population included fifteen male and two female carriers. Seventy healthy subjects (56 males and 14 females) were enrolled as normal controls. The study was initiated after obtaining the approval of the Institutional Ethics Committees, All India Institute of Medical Sciences (AIIMS), Lady Hardinge Medical College and Vallabhbhai Patel Chest Institute, Delhi and written informed consent was taken from all individuals (parents in case of children) participating in the study. The diagnosis of the X-ALD patients was based on clinical assessment, MRI and VLCFA analysis. Further categorization of the patients in different phenotypes of X-ALD was done according to the age of onset of the disease and the organ principally affected. The neurological examination was geared towards detection of behavioral disturbances, impaired cognition, and dementia in symptomatic patients, and included evaluation of higher mental function, cranial nerve, motor and cerebellar system function. Neurophysiologic examination included motor nerve conduction, F-wave and sensory nerve conduction studies. Phenotype specific T2 signal hyperintensity MRI characteristic patterns were observed in different X-ALD patients. All patients included in our study had elevated plasma levels of VLCFA particularly C26:0/C22:0 and C24:0/C22:0 ratios.

### Molecular Genetic Analysis

**Figure 1:**
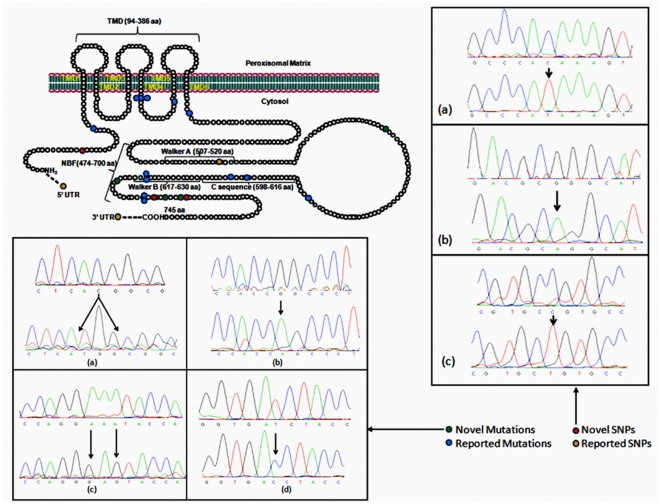
Mutations and SNPs identified in the ABCD1 gene. The upper left panel depicts all the mutations and SNPs that are localized in the schematic molecular structure of ALDP in the present study. The lower left panel shows the four novel mutations (a) c.1903_04insCCA/Val635delinsAlaMet (TGG in antisense strand with reverse primer), (b) c.1979G>A/Arg660Gln, (c) c.1993_95delinsGAG/Lys665delinsGlu and (d) c.1673T>C/Ile558Thr. The right panel shows the three novel SNPs (a) 476C/T (His30His), (b) 1950G/A (Ala650Ala) and (c) -32C/T in IVS9.

Peripheral blood (10 ml) was drawn from all patients (ccALD and AdolCALD) and their mothers, in sterile EDTA vacutainers (Greiner bio-one international, Germany). Genomic DNA was isolated from the blood leukocytes, using phenol-chloroform protocol [26]. All the 10 exons of *ABCD1* gene, including the flanking region of each exon (exon-intron boundaries), were amplified by polymerase chain reaction. The primers for amplification of first six exons (Table 1) of *ABCD1* gene were designed with DNASTAR (DNASTAR Inc., Madison, WI) software, and their specificity was confirmed by the Basic Local Alignment Search Tool in the Human Genome Database at the National Centre for Biotechnology Information (Bethesda, MD). M13-tailed primers were used for exons seven to ten due to the existence of paralogs copies on chromosome 2p11, 10p11, 16p11 and 22q11 [25], [27]. All exons were amplified in 50 µl volumes containing 10 m*M* Tris (pH 9), 50 m*M* KCl, 1.5 m*M* MgCl_2_, 0.01% gelatin, 0.2 m*M* each dNTP, 0.4 µ*M* each forward and reverse primers, 1.5 unit of Taq DNA polymerase (Bangalore Genei Pvt. Ltd., India), and 80 ng of genomic DNA template. PCR was performed for first six exons of *ABCD1* gene of by 35 cycles with the MJ Research Peltier Thermal Cycler (GMI Inc., Ramsey, MN)/Touchgene Gradient 200 (Techne Ltd., Cambridge, United Kingdom). Each cycle consisted of 30 s at 94°C for denaturation, 30 s at 56–62°C for annealing (depending upon the primers sets used), 30 s at 72°C for extension, and 10 min for final extension at 72°C. The amplification of exons 7 to 10 were performed in addition to 0.5 *M* betaine with PCR conditions as 94°C for 5 min, 35 cycles of 94°C for 45 s and 70°C for 1 min. Polymerase chain reaction amplicons were purified by removing excess primers and nucleotides, by using DNA isolation kit (Biological Industries, Kibbutz Beit Haemek, Israel). Direct sequencing of the *ABCD1* gene of X-ALD patients and control samples were performed using ABI 3100 DNA automated sequencer with BigDye cycle sequencing ready reaction (Applied Biosystems Inc., Foster City, CA) (Figure 1, Supporting Information S1 and S2).

**Table 1:** Primers and cyclic conditions used for the amplification of the coding and intron-exons boundaries of *ABCD1*.

(Exon/s)*	Forward Primer (5′-3′)	Reverse Primer (5′-3′)	Annealing Temp. (°C)	Fragment Length (bp)
1a	GGAGGAGGAGAAGGTGGAGAGG	AGACGGCTGCGGAACGACA	64	834
1b	CCTCATCGCCCTCCCTGCTACCTT	GCCTCCCTGCCACACGCTTCC	64	538
2	GGCCCCACCCAATCGTAAC	CAGCGGTATGGGGGACAGG	56	392
3 & 4	GCCTGTGATGTGCTCTGGGTTGGT	TGAGTGAGGGAGCGGGAATAG	62	704
5	CCAGGGGCACGCAGACTC	GGAACGCCGGGGTGAAC	60	408
6	CAGGAGGCCATAGGGTACGGGAA	GCAGTGGATCGGGCGGGTTTG	56	372

*Exon one was amplified into two amplicons 1a and 1b due to its large size while exon 3 and 4 are amplified into a single amplicon due to their relatively small size. Exon 7–10 were amplified by M13 tailed primers^25^.

### PBMC cell lysis

Peripheral blood mononuclear cells (PBMC) were isolated from whole blood using ficoll density gradient centrifugation at 670×g for 30 minutes. Cells were washed with 1xPBS and cell pellet lysed in ice-cold 100–200 µl of radioimmunoprecipitation assay (RIPA) buffer with protease inhibitors and supernatant was stored at −80°C.

### Western blot analysis

Total protein contents were determined with Pierce BSA total protein assay kit (Thermo Fisher Scientific Inc., Pierce Protein Research Products, Rockford, IL USA). Equal amount of protein extract (150 µg of total protein per lane) were loaded on 12% tris-glycine gradient SDS-polyacrylamide gel with Amersham full range Rainbow protein molecular weight marker (GE Healthcare UK Limited Amersham Place, Buckinghamshire, UK), electrophoresed and transferred to nitrocellulose membrane (Advanced Microdevices Pvt. Ltd, Ambala Cantt, India). Membranes were blocked with 5% blotto, non fat dry milk powder (Santa Cruz Biotechnology Inc., Santa Cruz, CA. USA) in TBST (20 m*M* Tris; pH 7.5, 500 m*M* NaCl, 0.05% Twee 20). Membrane was incubated with primary antibody mouse anti-ALDP monoclonal antibody, MAB2162 (1∶500; Chemicon International, Millipore, Billerica, MA USA). A rabbit anti-mouse horseradish peroxidase conjugated secondary antibody (Cell Signaling Technology Inc., Danvers, MA USA) was used at a dilution of 1∶2000 and detected with Amersham ECL Advance western blotting detection kit (GE Healthcare UK Limited Amersham Place, Buckinghamshire, UK).

## Results

### Clinical analysis

The study group consisted of 17 probands from different families: 15 male patients and 2 female carriers. Other novel mutations were reported earlier as case studies [28], [29] and uploaded in Human Genetics database [30]. The well available clinical details are summarized in Table 2. ccALD (Childhood cerebral adrenoleukodystrophy) was the most common phenotype in Indian population. The clinical features included neuroregression, seizures, deterioration of hearing, behaviour problem, cognitive decline, speech problem and abnormal gait. Cerebral MRI showed extensive demyelination in the parieto-occipital or frontal white matter. Manifestation of AdolCALD (Adolescent cerebral) and ACALD (adult cerebral adrenoleukodystrophy) are similar to ccALD, but the progression is somewhat slower. Two patients with AMN (adrenomyeloneuropathy) were included in our study group. The nerve conduction studies in both the patients indicated axonal polyneuropathy. MRI indicated internal capsule, basal ganglia and mesencephalon demyelination but no cerebral involvement. The patients had complaint of hypogonadism. The progression of the inflammatory response in spinal cord was slow. Two female carriers were identified on the basis of probands that died partially diagnosed. The heterozygous females were asymptomatic but had increased plasma VLCFA levels.

**Table 2:** Neurological, radiological, biochemical and other clinical examination within different phenotypes of adrenoleukodystrophy.

	ccALD^1^	AdolCALD^2^	ACALD^3^	AMN^4^	A. Females
**Relative frequencies (%)**	47	17	12	12	12
**Age of onset**	<10	11.–21	>21	>19	At any age
**Behavioural disturbances**	+	+	Present in few	-	-
**Impaired Cognition**	+	+	+	-	-
**Dementia**	-	Less frequent	+	-	-
**Polyneuropathy**	-	-	-	Axonal	-
**Impaired endocrine symptom**	Less frequent	Appear in few	Appear in most	+	-
**Hypogonadism**	-	-	-	+	Not observed
**Neuropsychological problem**	+	+	+	-	-
**Progression**	Very rapid	Rapid	Rapid	Slow	No sign
**Cerebral MRI Abnormality**	Parieto-occipital	Fronal, parieto- occipital	Occipital,corticospinal	Basal ganglia mesencephalon	Absent
**Enhanced VLCFA in plasma**	+	+	+	+	+

1 ccALD-Childhood Adrenoleukodystrophy,

2 AdolCALD- Adolescent cerebral Adrenoleukodystrophy,

3 ACALD-Adult Cerebral Adrenoleukodystrophy,

4 AMN-Adrenomyeloneuropathy. A.-Asymptomatic, (-) absent, (+) present.

### Mutational analysis

All mutations were present in the cytoplasmic domain of ALDP as represented schematically in figure 1 in our study. No mutation was present in the peroxisomal matrix. It is clear that each patient possessed only one mutation in the *ABCD1* gene (Table 3). In all, there were 13 different mutations in 17 patients [Figure 1 and Supporting Information S1 (a–i)]. Further, 10 non-recurrent and 3 recurrent mutations in 7 patients were present in Indian population. Of these, 8 were missense (10 patients), 3 were frameshift (5 patients) and 2 insertion/deletion (2 patients). One common recurrent missense mutation c.796G>A (Gly266Arg) in exon 1 was present in three patients from unrelated families with different phenotypes. The remaining 2 recurrent mutations were frameshift, g.1866-10G>A (Agr622fs) identified in IVS8 (intervening sequence 8) and c.1939_40insGG (Ala646fs) in exon 9, each type was observed in two different unrelated patients separately.

**Table 3:** Molecular analysis of 17 patients with X-linked adrenoleukodystrophy syndrome.

Patients	Phenotype^1^	Age(Year)	Sex	Exon/IVS	Mutation Type	Mutations	Protein Localization	ALDP	PSIC Score^5^
**P01** *	ccALD	4	M	9	Inframe del/ins	c.1903_04delinsCCA/Val635delinsAlaMet	NBF	+ +	-
**P02** *	ccALD	5	M	9	Missense	c.1979G>A/Arg660Gln	-	-	2.409
**P03**	ccALD	3	M	IVS8^4^	Frameshift	g.1866-10G>A/Arg622fs	Walker B^3^	-	-
**P04**	ccALD	4.5	M	1	Missense	c.796G>A/Gly266Arg	TMD	+ ++	2.539
**P05**	ccALD	6	M	9	Frameshift	c.1939_40insGG/Ala646fs	NBF	n.d	-
**P06**	ccALD	7	M	2	Missense	c.904G>A/Glu302Lys	TMD	+ +	2.194
**P07**	ccALD	8	M	3	Missense	c.1202G>A/Arg401Gln	-	+ ++	2.396
**P08** *	ccALD	8	M	10	Inframe del/ins	c.1993_95delinsGAG/Lys665delinsGlu	-	+ +	-
**P09**	AdolCALD	11	M	1	Missense	c.796G>A/Gly266Arg	TMD	+ ++	2.539
**P10**	AdolCALD	11	M	8	Missense	c.1816T>C/Ser606Pro	C sequence	-	2.499
**P11**	AdolCALD	15	M	IVS8	Frameshift	g.1866-10G>A/Arg622fs	Walker B	+	-
**P12**	ACALD	42	M	8	Missense	c.1825G>A/Glu609Lys	C sequence^3^	-	2.075
**P13** *	ACALD	46	M	7	Missense	c.1673T>C/Ile558Thr	NBF^3^	+ ++	1.211
**P14**	AMN	26	M	9	Frameshift	c.1939_40insGG/Ala646fs	-	-	-
**P15**	AMN	35	M	1	Missense	c.796G>A/Gly266Arg	TMD^2^	+ ++	2.539
**P16**	Asymptomatic	18	F	7	Missense	c.1771C>T/Arg591Trp	NBF	+ ++	2.818
**P17**	Asymptomatic	26	F	1	Frameshift	c.110_17del8/Val36fs	-	+ +	-

*Novel Mutations,

1 ccALD-Childhood Adrenoleukodystrophy, AMN-Adrenomyeloneuropathy, ACALD-Adult Cerebral Adrenoleukodystrophy, AdolCALD- Adolescent cerebral Adrenoleukodystrophy.

2 TMD: Transmembrane Domain.

3 The nucleotide-binding fold (NBF), with Walker A and B regions – separated by the C sequence.

4 IVS- Intervening Sequence,

5 PolyPhen **P**osition-**S**pecific **I**ndependent **C**ounts. Normal ALDP level (+++), Reduced ALDP level (++), Very low Level of ALDP (+), Absence of ALDP (−), n.d: not defined.

Of the 10 non-recurrent mutations in our study group i.e a frameshift mutation c.110_117del8 (Val36fs) in exon 1 was identified in an asymptomatic heterozygous female and 5 missense mutations, c.904G>A (Glu302Lys) in exon 2, c.1202G>A (Arg401Gln) in exon 3, c.1771C>T (Arg591Trp) in exon 7, c.1816T>C (Ser606Pro) and c.1825G>A (Glu609Lys) in exon 8 were present in 5 different patients. The remaining 4 non-recurrent mutations were novel which included 2 missense and 2 inframe amino acid deletion/insertion. The 2 missense novel mutations c.1673T>C (Ile558Thr) and c.1979G>A (Arg660Gln) were identified in exons 7 and 9 respectively. Inframe amino acid deletion/insertion mutation c.1903_04insCCA (Val635delinsAlaMet) and c.1993_95delinsGAG (Asn665delinsGln) were present in exon 9 and 10 respectively.

The possible impact of missense mutations on the structure and function of protein was predicted by Position-Specific Independent Counts (PSIC) using Polymorphism Phenotyping (PolyPhen) tool. High PSIC score difference (>0.5) indicates more damaging effect of the substituted amino acid (http://genetics.bwh.harvard.edu/pph/). Most of the missense mutations were predicted to have deleterious effect on ALDP in our study (Table 3).

### Protein expression analysis

**Figure 2:**
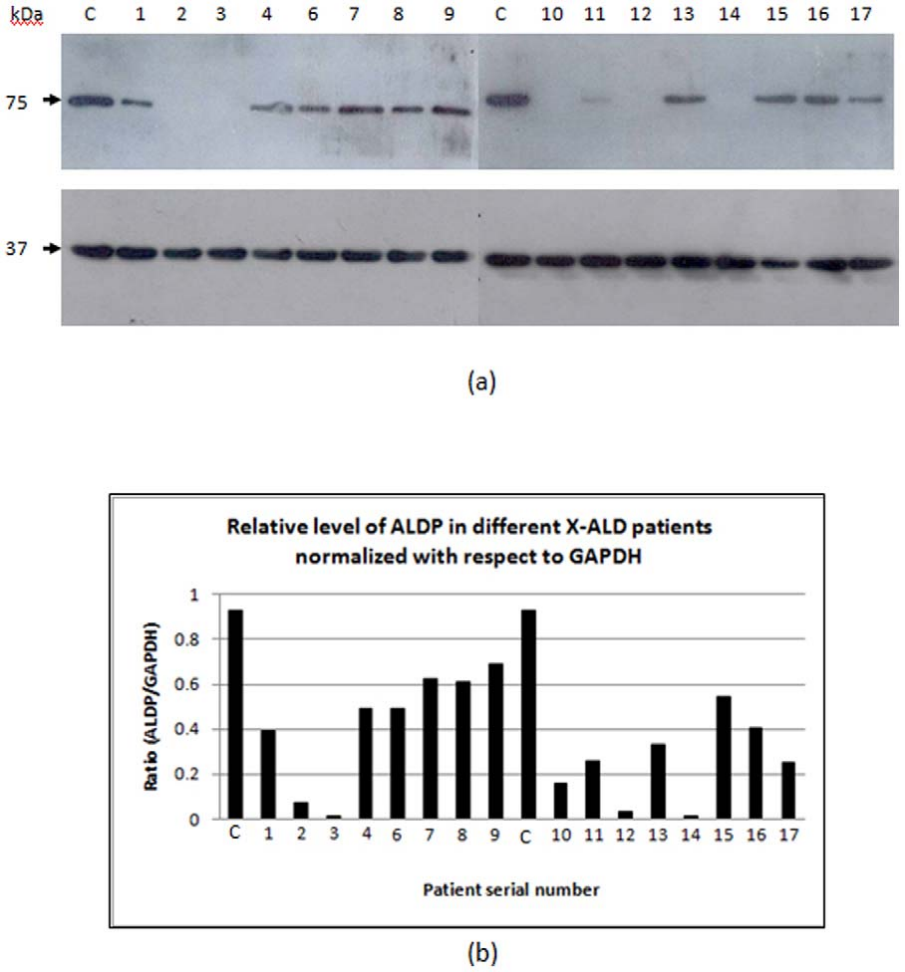
ALDP expression level in peripheral blood mononuclear cells (PBMCs) in different patients. (a) Top Panel shows the western blot of the patients ALDP level (except patient number P05) in total PBMCs lysate examined with mouse anti-ALDP monoclonal antibody as explained in material and method. Lane 1 and 10 (Control by symbol “C”), Lane 2 (P01, ccALD, V635delins A & M), Lane 3 (P02, ccALD, R660Q), Lane 4 (P03, ccALD, R622fs), Lane 5 (P04, ccALD, G266R), Lane 6 (P06, ccALD, E302K), Lane 7 (P07, ccALD, R401Q), Lane 8 (P08, ccALD, K665delinsE), Lane 9 (P09, AdolCALD, G266R), Lane 11 (P10, AdolCALD, S606P), Lane 12 (P11, AdolCALD, R622fs), Lane 13 (P12, ACALD, E609K), Lane 14 (P13, ACALD, I558T), Lane 15 (P14, AMN, A646fs), Lane 16 (P15, AMN, G266R), Lane 17 (P16, asymptomatic female, R591W) and Lane 18 (P17, asymptomatic female,). The lower panel shows the GAPDH as the loading control for each lane. (b) Densometric quantification of the ALDP in X-ALD patients normalized to GAPDH. The number along the x-axis indicated the patient serial number and symbol “C” represents healthy control.

The ALDP expression level was detected by western blot with mouse anti-ALDP monoclonal antibody that bind to 495–648 fragment as a fusion protein. Its expression was found to be either absent or decreased in all the patients (Figure 2). ALDP level in one of the patients (number P05) could not be determined due to insufficient availability of the sample. ALDP level was not detectable in five patients (P02, P03, P10, P12 and P14). We observed around fifty percent reduction in six patients (P01, P04, P06, P13, P15 and P16) and much more in another two patients (P11 and P17), (Figure 2). The remaining three (P07, P08 and P09) showed intermediate level of ALDP. The variation in level of proteins is random and not found to be associated with severity or the phenotype of the disease. However, all patients with framshift mutation showed relatively more decreased or total absence of ALDP. Two patients containing the same mutation in the intervening sequence (g.1866-10G>A/Arg622fs) showed different pattern (totally absent in P03 while very mild expression in P11) of ALDP. Interestingly, we found reverse trend of ALDP presence (12/17 = 0.70) in our population as compared to other population.

### Polymorphism analysis

In *ABCD1* gene, a total of six SNPs were identified, out of them three were novel (Table 4). Two novel SNPs, 90C/T (His30His) in exon 1 and 1950G/A (Ala650Ala) in exon 9 were observed, one each in unrelated healthy and patient samples. 1992-32C/T in IVS9 was identified in two controls and one X-ALD patient sample. The frequently occurring reported exonic SNP (rs41314153) was located in exon 6 of the *ABCD1* gene 1548G/A (Leu516Leu) with MAF (minor allele frequency) “T” 16/84 (19.04%) in healthy normal controls and 3/20 (15%) in adrenoleukodystrophy patients. Two other frequently occurring SNPs viz. -59C/T (rs4148030) and 2238+8C/G (rs2229539), were identified in 5′ and 3′ UTR respectively [(Table 4 and supporting information S2 (a–c)]. The MAF “T” of −59C/T was observed 17.85% (15/84) and 20% (4/20) while MAF “G” of 2238+8C/G was 28.57% (24/84) and 35% (7/20) in controls and patients respectively.

**Table 4:** SNP analysis in *ABCD1* gene in X-linked adrenoleukodystrophy cases and healthy controls.

Allele	Gene Region	SNP	Controls (n = 70)	Patients (n = 17)	Status
**5′UTR (C/T)**	5′ UTR	−59C/T	T = 15/84 (17.85%)	T = 4/20 (20%)	Frequent
**His30His** *	Exon 1	90C/T	T = 1/84 (Unique)	T = 1/20 (Unique)	Unique
**Leu516Leu**	Exon 6	1548G/A	A = 16/84 (19.04%)	A = 3/20 (15%)	Frequent
**Ala650Ala** *	Exon 9	1950G/A	A = 1/84 (Unique)	A = 1/20 (Unique)	Unique
**IVS9 (C/T)** *	IVS 9	1992-32C/T	T = 2/84 (2.38%)	T = 1/20 (Unique)	Unique
**3′UTR (stop+8)**	3′ UTR	2238+8C/G	G = 24/84 (28.57%)	G = 7/20 (35%)	Frequent

*Novel SNPs.

Similar frequencies of different SNPs were observed in unrelated X-ALD patients and controls. A case-control association was not observed in general and further analysis did not reveal evidence of association of polymorphisms with different X-ALD phenotypes.

## Discussion

The present study describes the clinical and genetic analysis of 17 X-linked adrenoleukodystrophy patients. Four novel mutations were present in four unrelated patients. Three recurrent and ten non-recurrent mutations in our study group were detected in the *ABCD1* gene. Presence of one mutation in each patient indicates the significance of *ABCD1* to be one of the most important candidate genes responsible for the adrenoleukodystrophy syndrome. No complete gene deletions or nonsense mutations were observed in Indian population. Of all 17 X-ALD patients, 10 possesed missense, 5 frameshift, and 2 inertion/deletion mutations and all are present in the cytoplasmic domain of ALDP. We have uploaded the mutations in the X-ALD database (http://www.x-ald.nl/). The frequency of missense mutations in our population is comparable to the X-ALD in other populations, most of which result in the lower steady-state levels of ALDP (Figure 2). However, no systematic study is present to support the predicted structure and function of ALDP which is expressed by *ABCD1* gene [31]. The effects of alteration of a single amino acid on the biochemical role performed by this protein are difficult to infer, but any missense mutation leading to decreased levels of ALDP (while there is no effect on the levels of *ABCD1* transcript) may interfere with the peroxisomal targeting mechanisms of the newly synthesized ALDP molecules and their correct membrane insertion or their correct folding. Thus, most of the mutations identified in our study might also interfere with any of these processes. However, the possibility of degradation due to misfolding of ALDP cannot be ruled out. We argue that the total absence of ALDP in 5 patients supports this hypothesis. The remaining patient showed reduced levels of ALDP as compared to the control. Moreover, the PSCI score difference clearly predicted their deleterious effect on ALDP (Table 3).

In June 2011, 1223 mutations in the *ABCD1* gene, of which 574 (47%) are non-recurrent mutations, have been identified and listed in X-ALD database (http://www.x-ald.nl/). They have been described for all the 10 exons of the gene, and are generally infrequent and usually confined to a single family. The majority of X-ALD patients in our study group had non-recurrent (59%) mutations. Single base pair substitution or point mutations represented the majority (70%) of mutations. The remaining was deletion/insertion of two or more nucleotides. All the point mutations identified in the *ABCD1* gene were transition mutations. Most of the mutations (63%) were present in the functionally relevant sites viz. transmembrane and ATP-binding domain of the ALD protein which indicates that X-ALD mutations are not distributed uniformly in the *ABCD1* gene. This observation supports the contention that higher frequencies of disease causing mutations are expected to be present in regions of the protein that are crucial for its functions. Therefore, the gene analysis of the X-ALD patients should initially include screening for mutations in the functionally relevant region, and then the other region of the gene by directsequencing. It is well known that geographical distributions of ethnicity of patients have profound effects on mutations. In some ethnic groups as in whites, exon 5 was the possible hot-spot segment, while in Chinese population it was exon 6. However in our population, we did not find any mutation in these regions [32]–[34]. Thus, a definite hot-spot mutation in Indian patients could not be observed, although c.796G>A (Gly266Arg) mutation in the transmembrane domain in exon 1 was present in three unrelated patients of different phenotypes. Interestingly, a novel intronic SNP 1992-32C/T was also observed in IVS9 in the patient with ccALD phenotype possessing c.796G>A (Gly266Arg). The significance of such SNPs is not known. However, the association of SNPs in linkage disequilibrium with a functional mutation that modifies the expression of this gene could not be excluded. We observed other two new exonic silent SNPs which were, 90C/T (His30His) in exon 1 and1950G/A (Ala650Ala) in exon 9 in two different unrelated ccALD patients (P04 and P07, Table 3). However, both of these patients show intermediate level of ALDP. The reports suggest that silent SNPs can have the effect on rate of translation from mRNA to protein [35]. The changes in the rate of translation, without any change in the amino acid sequence which could affect the protein structure and function, is intriguing and the mechanism in support of this contention is further needed to be understood. The lack of large number of common mutations with different ethnic groups and the private nature of *ABCD1* gene mutations in Indian population may be attributed to the biodiversity in the population.

The clinical course of X-ALD is unpredictable. Moreover no genotype (mutations)-phenotype (disease severity) correlation was observed in our study on Indian population. Our study further supports the fact that clinical manifestation of X-ALD is highly variable, there is no correlation of between clinical phenotype, VLCFA levels in plasma and geneotype, and the degree of loss of function of ALDP is not related to disease severity [36], [37]. This lack of correlation suggest that some other genetic factors might modulate the X-ALD phenotypes and several candidate genes have been suggested as potential modifiers [38], [39] or other yet unidentified factors [15]. However, none of these genes have a direct correlation with X-ALD phenotypes. Apart from this study, only few recent studies are available on adrenoleukodystrophy disease in India [28], [29], [40], [41]. Therefore, a more detailed investigation and analysis of this disease was obligatory to identify new significant polymorphism and mutations and the variability of allelic states in Indian Population. VLCFA level is at. borderline of normal in 0.1% of affected males and 15% obligate heterozygotes [42], but studies on mutation are the only reliable approach to determine the genetic status of each individual and to rule out the carrier status accurately. India has a multitude of genetically diverse, unadmixed and isolated population groups and large population size. These facts make India a fertile ground for gene-hunting and validation of results from genome-wide association and linkage studies. Our present study and further extended family screening will definitely facilitate the understanding of this disease more accurately which will help in early and more precise risk-prediction, as well as aid in further prevention and therapy.

## Supporting Information

Supporting Information S1 Frequently occurring mutations in (a) intervening sequence 8 (g.1866-10G>A/Arg622fs, shown as C>T in antisense strand) in P03 and P11, (b) exon 1 (c.796G>A/Gly266Arg, shown as C>T in antisense strand) in P04, P09 and P15, (c) exon 9 (c.1939_40insGG/Ala646fs, shown as CC in antisense strand) in P05 and P14, (d) exon 2 (c.904G>A/Glu302Lys) in P06, (e) exon 3 (c.1202G>A/Arg401Gln) in P07, (f) exon 8 (c.1816T>C/Ser606Pro) in P10, (g) exon 8 (c.1825G>A/Glu609Lys) in P12, (h) exon 7 (c.1771C>T/Arg591Trp) in P16 and (i) exon 1 (c.110_17del8/Val36fs) in P17.
(DOC)

Supporting Information S2 Frequently occurring single nucleotide polymorphisms (SNPs) in (a) 5′ UTR (-59C/T), (b) exon 6 (1548G/A, Leu516Leu) and (c) 3′ UTR (2238+8C/G) in *ABCD1* gene.
(DOC)
