# Supplementary figures and images for: Genomic Profiling Identifies Novel Mutations and SNPs in ABCD1 Gene: A Molecular, Biochemical and Clinical Analysis of X-ALD Cases in India
Source: PLoS One. 2011 Sep 22;6(9):e25094. doi: 10.1371/journal.pone.0025094 (PMC3178599; doi:10.1371/journal.pone.0025094)

**Mutations in *ABCD1* gene**


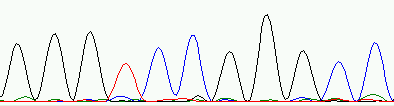


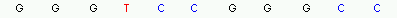


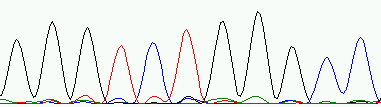


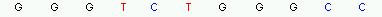


**(a)**


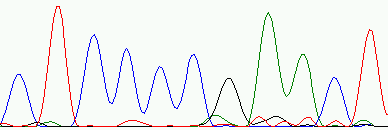


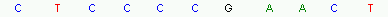


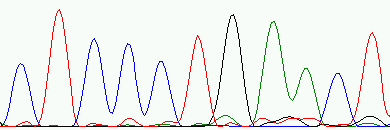


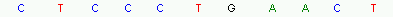


**(b)**


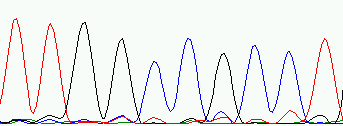


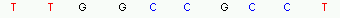


}


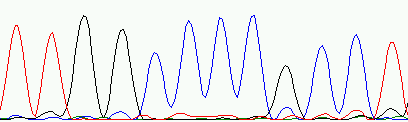


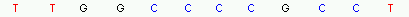


**(C)**


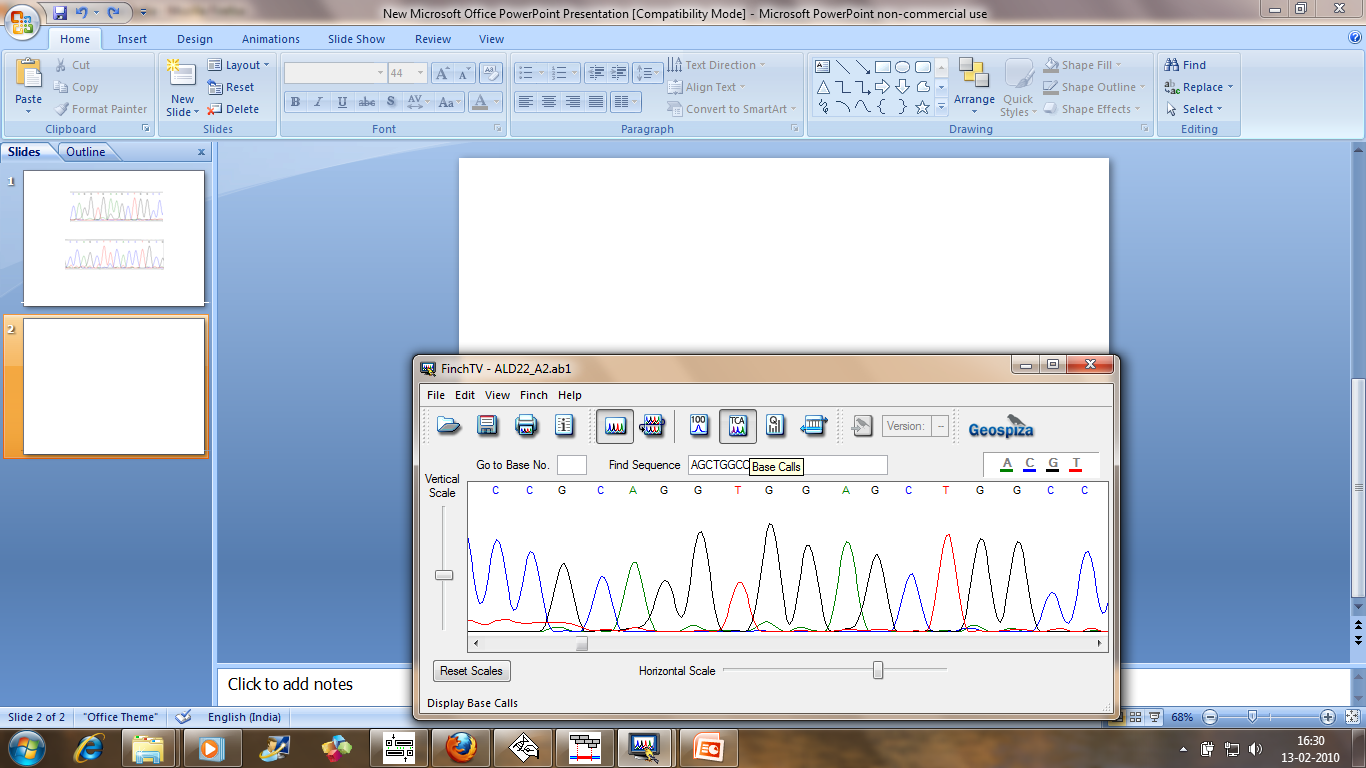


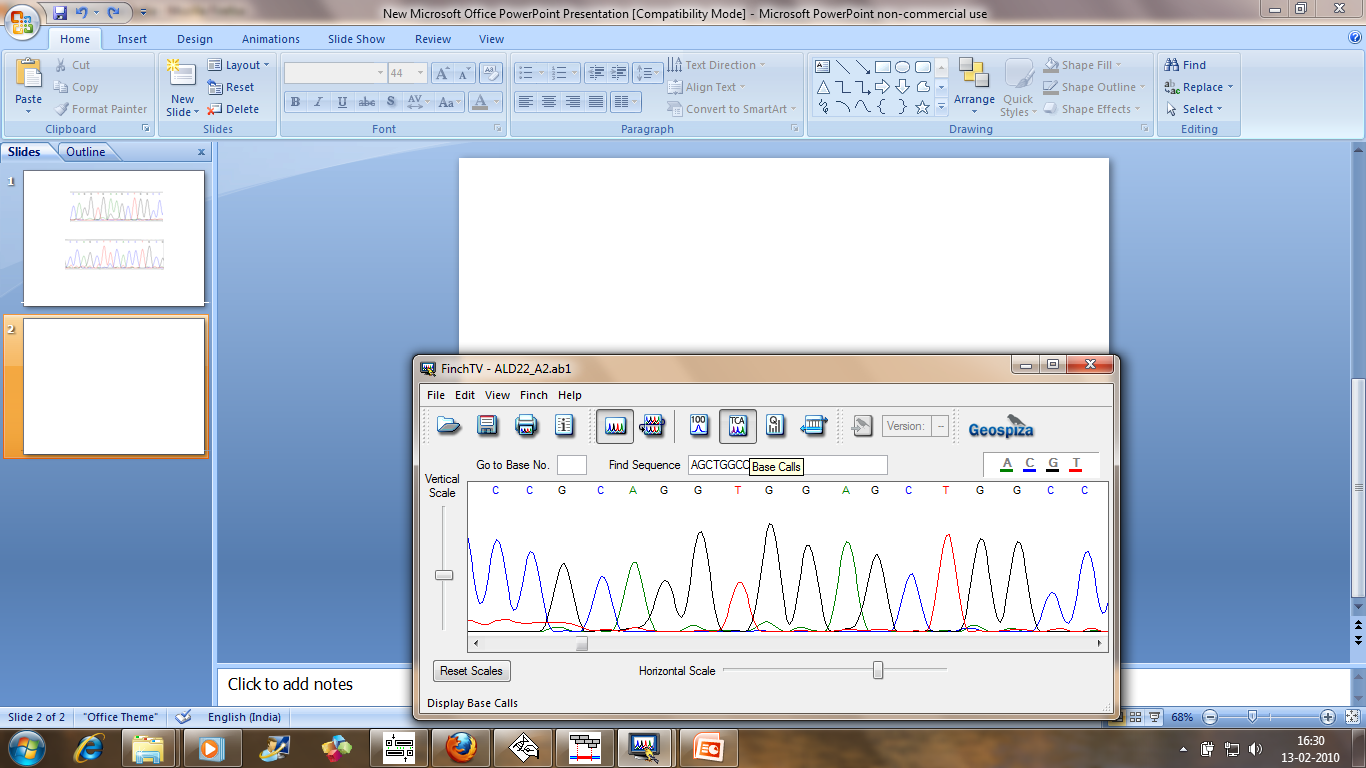


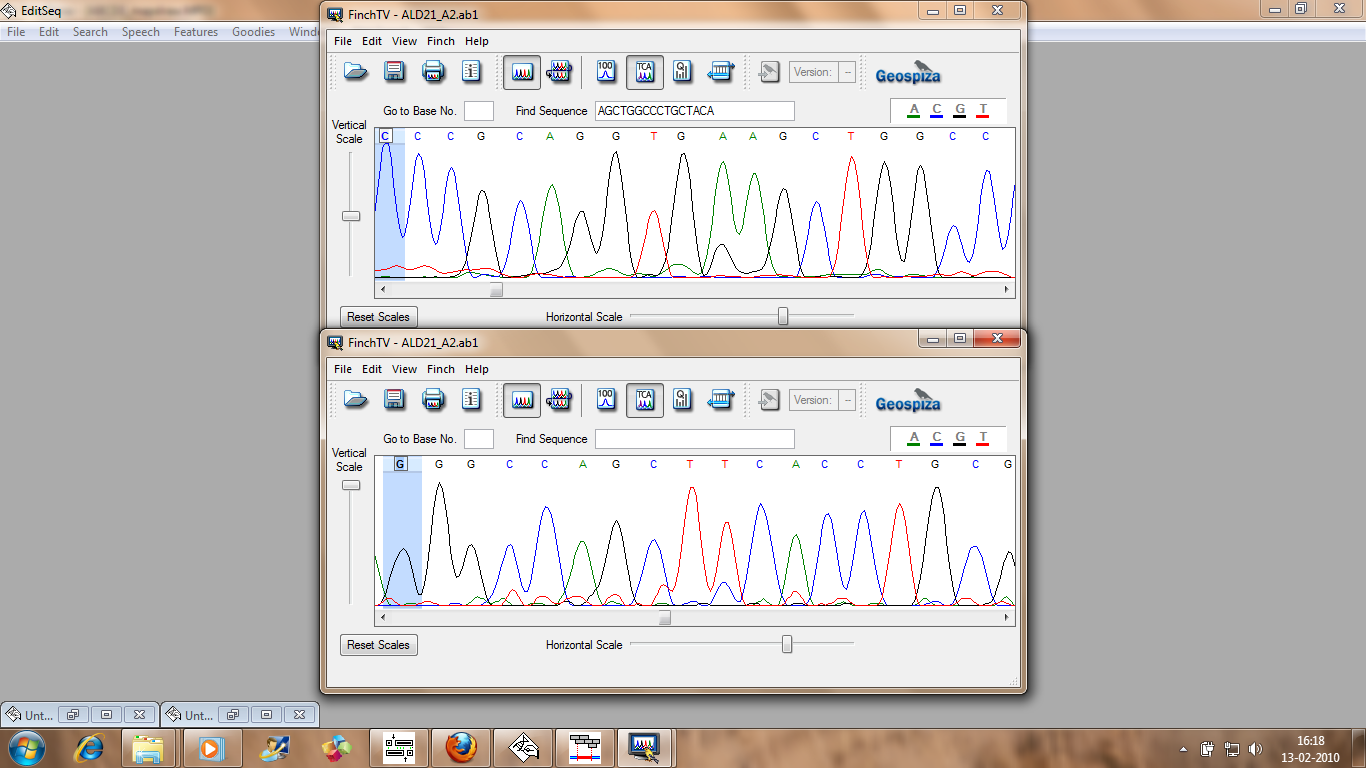


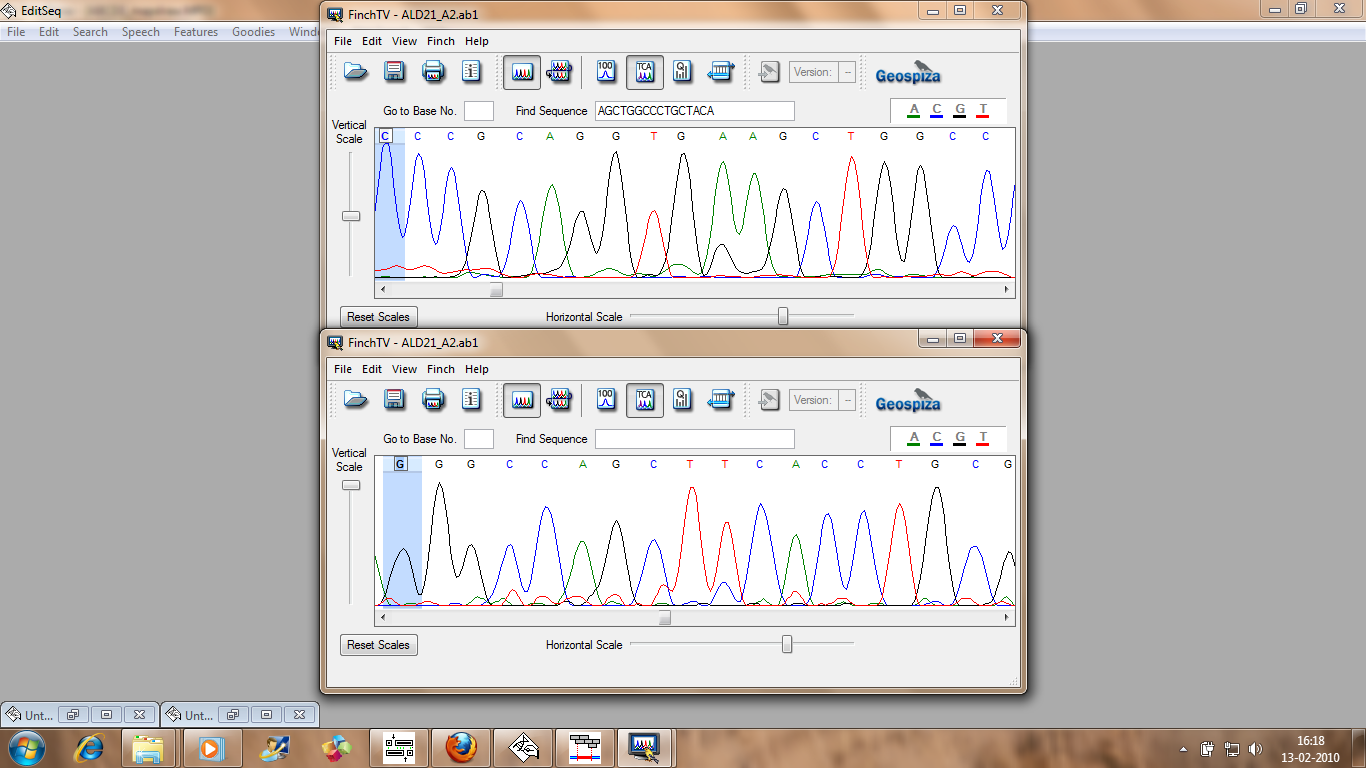


**(d)**


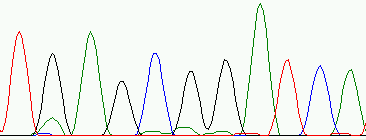


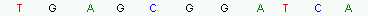


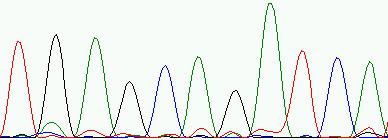


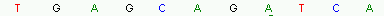


**(e)**


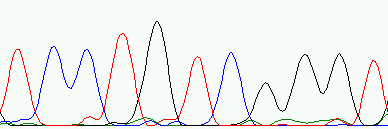


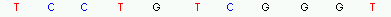


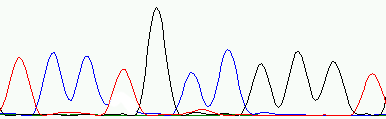


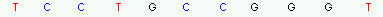


**(f)**


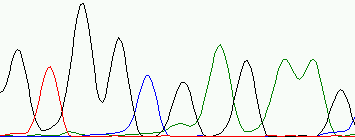


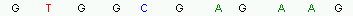


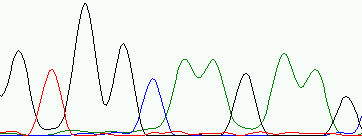


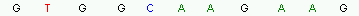


**(g)**


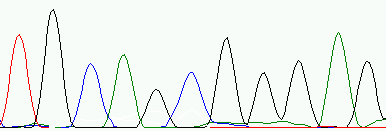


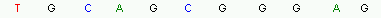


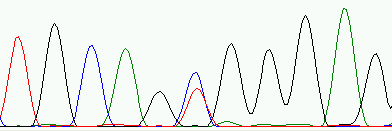


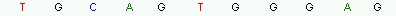


**(h)**


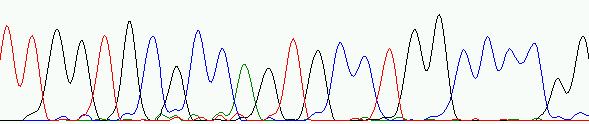


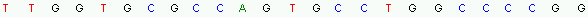


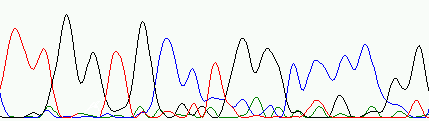


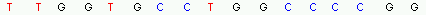


**(i)**

Supplement: Supporting Information S1 — Frequently occurring mutations in (a) intervening sequence 8 (g.1866-10G>A/Arg622fs, shown as C>T in antisense strand) in P03 and P11, (b) exon 1 (c.796G>A/Gly266Arg, shown as C>T in antisense strand) in P04, P09 and P15, (c) exon 9 (c.1939_40insGG/Ala646fs, shown as CC in antisense strand) in P05 and P14, (d) exon 2 (c.904G>A/Glu302Lys) in P06, (e) exon 3 (c.1202G>A/Arg401Gln) in P07, (f) exon 8 (c.1816T>C/Ser606Pro) in P10, (g) exon 8 (c.1825G>A/Glu609Lys) in P12, (h) exon 7 (c.1771C>T/Arg591Trp) in P16 and (i) exon 1 (c.110_17del8/Val36fs) in P17. (DOC) [file pone.0025094.s001.doc]

**SNPs in *ABCD1* gene**


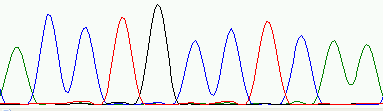

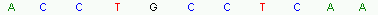


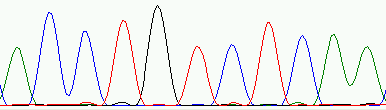


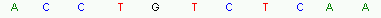


**(a)**


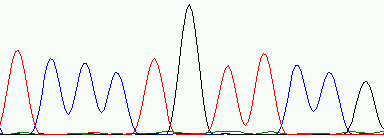


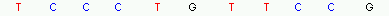


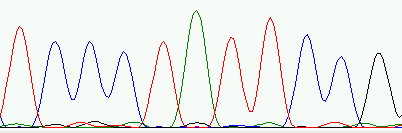


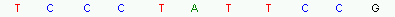


**(b)**


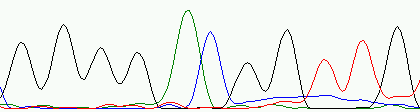


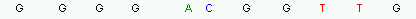


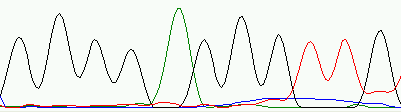


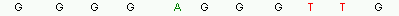


**(c)**

Supplement: Supporting Information S2 — Frequently occurring single nucleotide polymorphisms (SNPs) in (a) 5′ UTR (-59C/T), (b) exon 6 (1548G/A, Leu516Leu) and (c) 3′ UTR (2238+8C/G) in ABCD1 gene. (DOC) [file pone.0025094.s002.doc]
